# Supplementary material for: MetaRibo-Seq measures translation in microbiomes
Source: Nat Commun. 2020 Jun 29;11:3268. doi: 10.1038/s41467-020-17081-z (PMC7324362; doi:10.1038/s41467-020-17081-z)
Supplement: Supplementary file 10 — Supplementary Data 7 [file 41467_2020_17081_MOESM10_ESM.zip › File2/Confidence_High_Taxonomy/110115_out.krona.html]

Javascript must be enabled to view this page.

members
magnitude
magnitudeUnassigned
count
unassigned
taxon
rank

110115\_out

2

superkingdom
2
2

phylum
1
32066

1
203490
class

order
1
203491

1
1129771
family

32067
1
genus

species
1

SRS147151\_contig\_number\_18803
157687

1239
1
phylum

class
1
186801


SRS013940\_contig\_number\_contig-100\_29343.29344
186802
1
order
